# Fission yeast kinesin-8 controls chromosome congression independently of oscillations

**DOI:** 10.1242/jcs.160465

**Published:** 2015-10-15

**Authors:** Hadrien Mary, Jonathan Fouchard, Guillaume Gay, Céline Reyes, Tiphaine Gauthier, Clémence Gruget, Jacques Pécréaux, Sylvie Tournier, Yannick Gachet

**Affiliations:** 1 Université de Toulouse, LBCMCP, 118 route de Narbonne, Toulouse F-31062, France; 2 CNRS, LBCMCP-UMR5088, Toulouse F-31062, France; 3 DAMCB, 43 rue Horace Bertin, Marseille 13005, France; 4 IGDR, Institute of Genetics and Development of Rennes, University Rennes 1, Rennes F-35043, France

**Keywords:** Fission yeast, Mitosis, Kinesin-8, Kinetochore alignment

## Abstract

In higher eukaryotes, efficient chromosome congression relies, among other players, on the activity of chromokinesins. Here, we provide a quantitative analysis of kinetochore oscillations and positioning in *Schizosaccharomyces pombe*, a model organism lacking chromokinesins. In wild-type cells, chromosomes align during prophase and, while oscillating, maintain this alignment throughout metaphase. Chromosome oscillations are dispensable both for kinetochore congression and stable kinetochore alignment during metaphase. In higher eukaryotes, kinesin-8 family members control chromosome congression by regulating their oscillations. By contrast, here, we demonstrate that fission yeast kinesin-8 controls chromosome congression by an alternative mechanism. We propose that kinesin-8 aligns chromosomes by controlling pulling forces in a length-dependent manner. A coarse-grained model of chromosome segregation implemented with a length-dependent process that controls the force at kinetochores is necessary and sufficient to mimic kinetochore alignment, and prevents the appearance of lagging chromosomes. Taken together, these data illustrate how the local action of a motor protein at kinetochores provides spatial cues within the spindle to align chromosomes and to prevent aneuploidy.

## Abstract (precis)

**Highlighted Article:** Quantitative analysis in *S. pombe* reveals that chromosome oscillations are dispensable for kinetochore congression in mitosis. Kinesin-8 controls chromosome congression through length-dependent pulling forces.

## INTRODUCTION

Chromosome congression is an evolutionary conserved feature of mitosis thought to promote faithful segregation of sister chromatids into daughter cells (Kops et al., 2010). In higher eukaryotes, efficient chromosome congression relies on multiple mechanisms and involves several microtubule (MT)-dependent motor proteins. On chromosome arms, the chromokinesins create the polar ejection forces (PEFs) that allow chromosome congression (Antonio et al., 2000; Funabiki and Murray, 2000; Rieder et al., 1986; Wandke et al., 2012). A second mechanism of congression involves CENP-E at kinetochores mediating the sliding of chromosomes along spindle MTs (Cai et al., 2009; Kapoor et al., 2006). In addition to chromosome congression, bi-oriented sister kinetochores undergo oscillatory movements between the two spindle poles before chromosome segregation (Amaro et al., 2010; Pearson et al., 2001; Skibbens et al., 1993). Oscillations and congression of chromosomes are complex movements that require spatio-temporal control of tensional forces and attachment at the level of kinetochores that is far from being understood (Dumont and Mitchison, 2009). Multiple actors are involved in these processes, such as kinetochore components, non-kinetochore forces such as PEFs, motor proteins and MT-associated proteins, which control MT dynamics (Civelekoglu-Scholey et al., 2013; Joglekar et al., 2010; McIntosh, 2012). However, chromosome oscillations are not absolutely required for the execution of mitosis given that several cell types seem to successfully segregate chromosomes in the absence of oscillations (Desai et al., 1998; LaFountain et al., 2001).

Among the different players controlling kinetochore congression and oscillation, kinesin-8 family members are emerging as one of the most important motor proteins that participate in the correct distribution of forces within the spindle. Kinesin-8 is a highly processive motor known to regulate MT dynamics thanks to its plus- end-directed motility and plus-end-specific destabilizing activity (Gupta et al., 2006; Mayr et al., 2007). A combination of these two activities has been suggested to mediate an MT-length-dependent mechanism that is responsible for cellular MT-length homeostasis (Foethke et al., 2009; Tischer et al., 2009; Varga et al., 2006, 2009). Seminal works of West et al*.* and Garcia et al*.* have attested that Klp5 and Klp6, the fission yeast homologs of kinesin-8, are also required for normal chromosome movement and attachment (Garcia et al., 2002a; West et al., 2002). Interestingly, no chromokinesin homolog is present in fission yeast (Wood et al., 2002), so there are supposedly no anti-poleward ejection forces. There is also no poleward flux of tubulin within the spindle in this model system (Mallavarapu et al., 1999) as opposed to in higher eukaryotes (Mitchison, 1989). Thus, *Schizosaccharomyces pombe* has several features that make it a simple and attractive model to study the respective role of kinetochore components or MT dynamics in chromosome congression and oscillation. Mitosis in *S. pombe* consists of three phases (Nabeshima et al., 1998; Tatebe et al., 2001). During phase 1, a short (<2.0 μm) spindle is formed (prophase). In phase 2 (pro-metaphase, metaphase and anaphase A), the spindle maintains roughly the same length and the kinetochores make frequent, rapid movements between the poles. At the end of phase 2, sister chromatids separate and move towards the SPBs during anaphase A (Tournier et al., 2004). In phase 3 (anaphase B), the spindle elongates along the longitudinal axis of the cell.

To obtain a quantitative understanding of chromosome segregation, mathematical models of metaphase chromosome dynamics have been developed over the past few years (Civelekoglu-Scholey and Cimini, 2014; Vladimirou et al., 2011). Most of these models analyze how various components of a force-balance system affect chromosome oscillation, congression or segregation (Brust-Mascher et al., 2004; Civelekoglu-Scholey et al., 2013, 2006; Courtheoux et al., 2009; Gay et al., 2012; Joglekar and Hunt, 2002; Paul et al., 2009). However, to date, there are no specific and quantitative descriptions of the mechanisms controlling fission yeast chromosome alignment or oscillation in mitosis or their role in kinetochore attachment.

Here, we present a quantitative analysis describing chromosome positioning and oscillation in wild-type fission yeast cells. Fission yeast chromosomes align during prophase and, while oscillating, remain aligned throughout metaphase. We demonstrate that chromosome oscillation is dispensable for kinetochore congression. Importantly, the role of kinesin-8 in chromosome congression is independent from its function in restricting chromosome oscillations to the spindle midzone. Finally, *in silico* and *in vivo* evidence suggest that a length-dependent process could participate in chromosome congression in fission yeast.

## RESULTS

### Kinesin-8 actively controls and maintains kinetochore alignment during mitosis

**Fig. 1.**
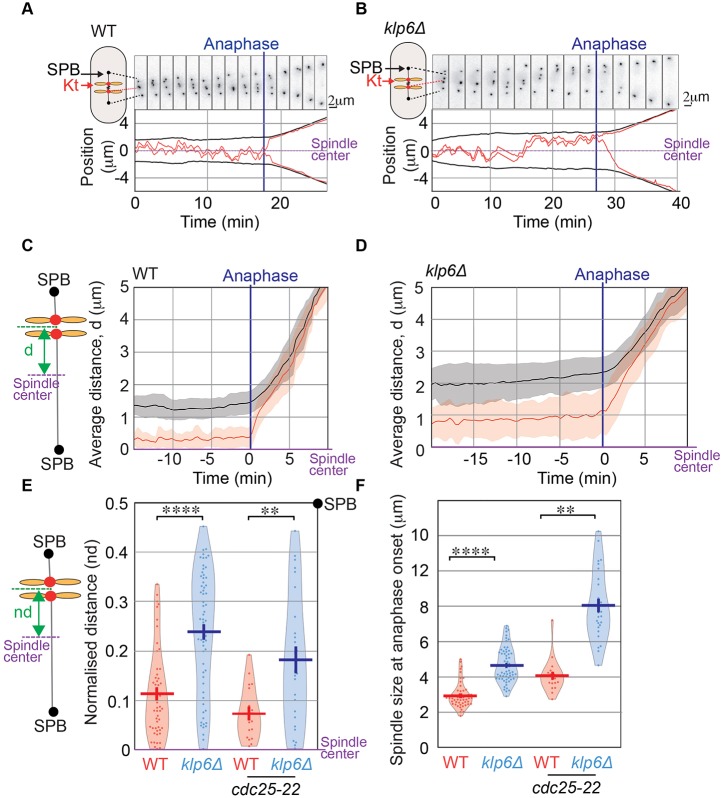
**The stability of sister kinetochore centering during metaphase depends on kinesin-8 activity.** (A,B) Typical timelapse fluorescence images of wild-type (WT) or *klp6*Δ cells expressing Cen2–GFP (marking the centromeric region of chromosome 2) and Cdc11–GFP (marking the spindle poles) during metaphase and anaphase. Frames were taken every 10 s. The lower panel is showing the corresponding trajectories of Cen2 (red) and SPBs (black) projected on a 1D axis whose origin is the spindle center. (C,D) Mean positioning of centromere 2 as a function of time from metaphase to anaphase. For each time-point, the absolute distance between Cen2 (red line) and the spindle center (purple line, 0 on the *y*-axis) or between the poles (black line) and the spindle center was computed and averaged from multiple movies (WT, *n*=52; klp6Δ, *n*=63) of mitotic cells. Pink and gray intervals indicate the s.d. (E) Global distribution of the normalized distances (nd) between Cen2 to the spindle center at anaphase onset in wild-type (0.11±0.001, *n*=52), *klp6*Δ (0.24±0.001, *n*=63), *cdc25-22* (0.007±0.001, *n*=19) and *cdc25-22 klp6*Δ (0.18±0.003, *n*=26) cells. Each distance between sister kinetochores to the spindle center is normalized according to spindle size so that sister kinetochore position varies between 0 (spindle center) to 0.5 (spindle poles). (F) Spindle size at anaphase onset in wild-type (2.95±0.01 μm, *n*=52), *klp6Δ* (4.68±0.12 μm, *n*=63), *cdc25-22* (4±0.02 μm, *n*=19) and *cdc25-22 klp6*Δ (8±0.04 μm, *n*=26) cells. The plots in E and F are violin plots, which combine a standard box plot with a density trace. Each circle represents a value in the dataset, the horizontal bar represents the mean of the distribution and the vertical bar represents s.e.m. **P<0.01, ****P<0.001 (Student's *t*-test).

To explore the mechanisms controlling kinetochore alignment in *S. pombe*, we performed 3D live-cell imaging in wild-type and kinesin-8-deleted cells. We simultaneously imaged the pericentromere region of chromosome 2 (marked with Cen2–GFP) (Yamamoto and Hiraoka, 2003) and the spindle pole bodies (SPBs) (Cdc11–GFP) (Tournier et al., 2004) during mitosis. The observed trajectories confirmed that kinetochores of cells deleted for Klp6 were often found near the poles as opposed to kinetochores of wild-type cells, which were confined in the vicinity of the spindle center (Fig. 1A,B; Movie 1 and 2).

To characterize and quantitatively measure the chronology of kinetochore positioning from phase 2 (pro-metaphase and metaphase) to anaphase onset, we computed from multiple trajectories the average distance (*d*) between sister centromeres (red line) and the spindle center (purple line) as a function of time in parallel with spindle elongation (SPB position in black, Fig. 1C,D; see details in Materials and Methods and note that the graph is not normalized to spindle size). This measure provides an estimate of the mean gap between kinetochores and the spindle center throughout metaphase, whereas its standard deviation (pink zone) reveals the spreading of this measure. In both cell lines, the average gap between Cen2 signals (red) and the spindle pole (black) was unchanged throughout the metaphase process (starting from 1.5 µm spindles) until anaphase onset. Chromosome position was maintained throughout phase 2 suggesting that a stationary process controls kinetochore alignment during metaphase progression. In the absence of kinesin-8, kinetochores were constantly positioned further away from the spindle center and the large standard deviation suggests that they are widely spread over the entire spindle (Fig. 1B,D). Statistical analysis performed immediately before anaphase confirmed that sister kinetochores are found at an average distance from the spindle center of 0.113±0.01 for wild-type cells (*n*=52) and 0.239±0.01 for *klp6Δ* cells (*n*=63) (normalized distance relative to spindle size; Fig. 1E). Consistently with the heterodimeric association of Klp5 and Klp6, which is required for kinesin-8 function (Unsworth et al., 2008), both *klp5Δ* and *klp5Δ klp6Δ* double mutant showed the same deficiency in kinetochore centering (Fig. S1).

Given that kinesin-8-deleted cells exhibit larger spindles and a substantial metaphase delay as can be seen in Fig. 1B,F (Garcia et al., 2002b), we wondered whether these defects could indirectly be the cause of kinetochore mis-alignment. We tested the former hypothesis by increasing spindle size of both cell types using the temperature-sensitive cell cycle progression mutant *cdc25-22*. To increase spindle size, we arrested *cdc25-22* and *cdc25-22 klp6*Δ cells at the restrictive temperature for 3 h (Fig. 1E,F; Fig. S1D). Increasing spindle size in the *cdc25-22* background had no effect on kinetochore alignment (Fig. 1E,F). Thus, the increased outward pushing forces developed at the spindle midzone when kinesin-8 is absent are not the cause of kinetochore mis-alignment.

**Fig. 2.**
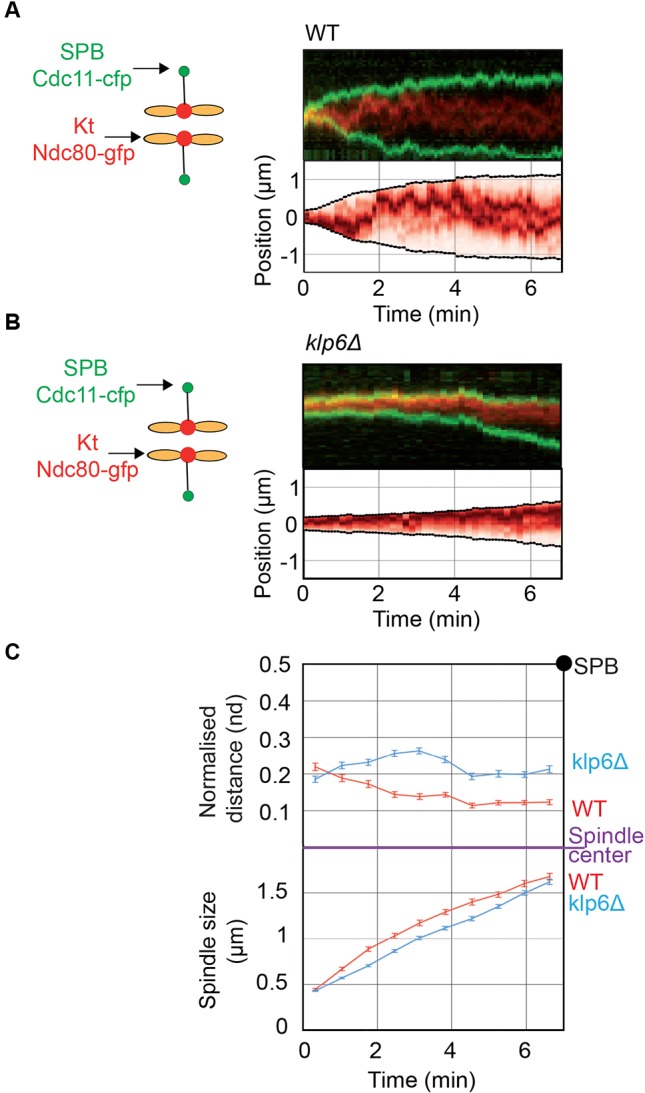
**Kinesin-8-dependent chromosome alignment is performed through an active process before metaphase.** (A,B) Upper panel. Typical kymographs of wild-type (WT) or *klp6*Δ cells expressing Ndc80–GFP (shown in red) and Cdc11–CFP (shown in green) from prophase to metaphase. Frames were taken every 5 s. The lower panel shows a computed kymograph where each time-point was normalized to the median intensity of the Ndc80 signal in the whole stack. (C) Upper panel; mean kinetochore distance to the spindle center normalized to spindle size (nd). The position of the maximum peak of intensity in the normalized kymographs (example in lower panel A and B) is used to identify the average position of the three chromosomes (assuming that they are close to each other in prophase). Data obtained from multiple kymographs of cells (*n*=27 for wild type; *n*=34 for *klp6*Δ) were used to plot the average position of kinetochores according to time. The time zero represents phase 1 onset; error bars are s.e.m. Note that the value of kinetochore positioning obtained at 4 min is very similar to that found in Fig. 1E. Lower panel; corresponding mean spindle size.

Given that wild-type chromosomes were correctly aligned in metaphase, we hypothesized that kinetochore centering might occur earlier, possibly during prophase. We thus followed the position of the six kinetochores (marked by *ndc80–GFP*) according to the spindle poles (marked by *cdc11–CFP*) from phase 1 to the onset of phase 2 by tracking the maximum intensity of Ndc80 signals over time (Fig. 2A,B). The average position of kinetochores obtained from multiple Ndc80 trajectories was plotted according to time (Fig. 2C). In wild-type cells, at the onset of spindle pole separation (spindles under 0.5 µm), kinetochores progressively aligned to reach their relative metaphase position at a spindle size of 1.2 μm (time 4 min; Fig. 2A,C). By contrast, in kinesin-8-deleted cells, kinetochores remained randomly distributed during phase 1, phase 2 and the onset of anaphase (Fig. 2B,C).

To conclude, our results suggest that kinetochore centering in fission yeast takes place very early in mitosis, prior to metaphase, is maintained until anaphase onset and requires the function of kinesin-8.

### Decreasing the amplitude of chromosome oscillation is not sufficient to correct kinesin-8-centering defects

**Fig. 3.**
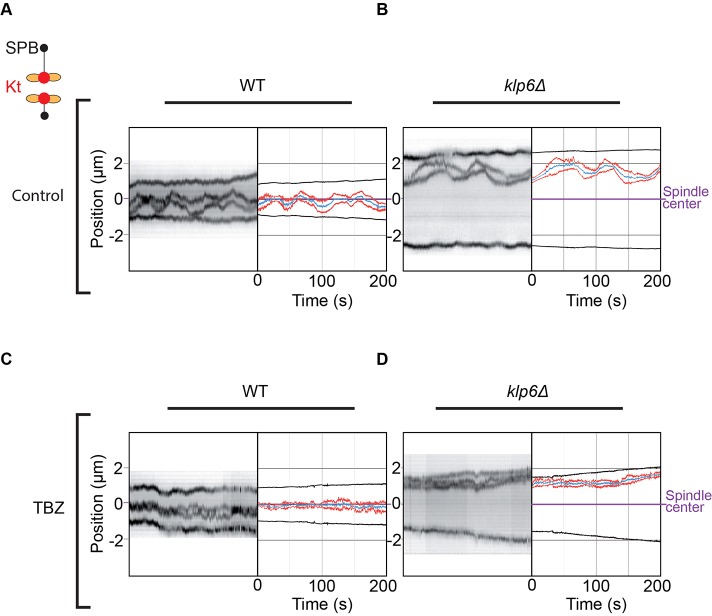
**Low doses of the MT-depolymerizing drug TBZ abolish chromosome oscillation but not centering.** (A,B) Typical kymographs obtained at high-frame rate (frames were taken every 0.1 s) and corresponding tracked trajectories of wild-type (WT) or *klp6*Δ cells expressing Cen2–GFP (sister centromeres in red and their mid position in blue) and Cdc11–GFP (SPBs, black) during metaphase. (C,D) Typical kymographs obtained at high-frame rate (frames were taken every 0.1 s) and corresponding tracked trajectories of wild-type or *klp6*Δ cells expressing Cen2–GFP (sister centromeres in red and their mid position in blue) and Cdc11–GFP (SPBs, black) during metaphase, in the presence of 10 µg/ml of TBZ.

Previous results obtained in higher eukaryotes have shown that the misalignment of kinetochores during metaphase correlates with larger and faster kinetochore movement in kinesin-8-deleted cells (Jaqaman et al., 2010; Stumpff et al., 2008). In order to accurately quantify chromosome movement in *S. pombe*, we imaged the centromeres of chromosome 2 (Cen2) and the spindle poles (Cdc11) at high frame rate (i.e. one image each 0.1 s) on limited segments of metaphase in wild-type and kinesin-8-deleted cells. Fig. 3A,B illustrates the typical trajectories obtained. To abolish kinetochore movement during metaphase, wild-type and kinesin-8-deleted cells were filmed in the presence of low doses of thiabendazole (TBZ; 10 µg/ml), a drug known to destabilize fission yeast interphase and spindle MTs when used at high doses (Umesono et al., 1983). In the presence of TBZ, the spindle size in phase 2 was similar to control conditions (Fig. S1C), suggesting that the global structure of the spindle is not affected by this treatment. However, an analysis of interphase MT dynamics in cells expressing *atb2–GFP* (tubulin–GFP) showed that the addition of TBZ reduces MT shrinkage rate and rescue frequency by a factor of two, whereas the growth rate and catastrophe frequency were not significantly affected by this treatment (Fig. S2).

**Fig. 4.**
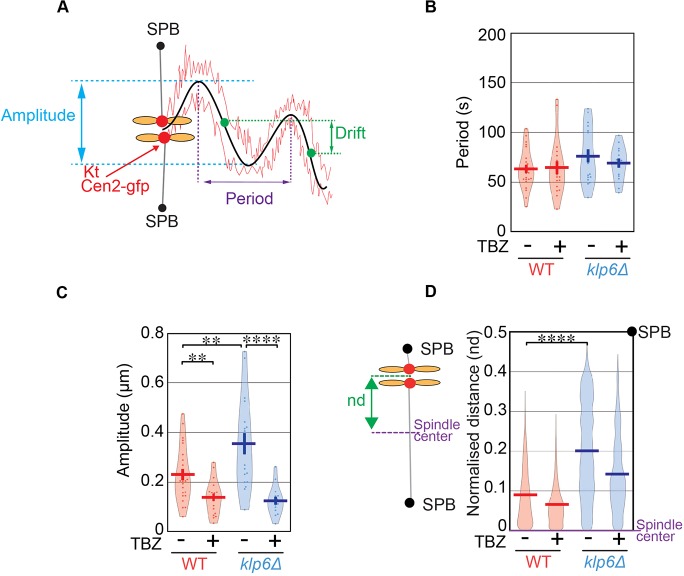
**Kinetochore oscillations are not required for the role of kinesin-8 in chromosome congression.** (A) Schematic representation of oscillation periods, amplitudes and drifts in kinetochore trajectories. (B) Periods of kinetochore oscillations obtained with the Fourier transform method during metaphase in wild-type (WT) (control, 63±4 s, *n*=24; TBZ, 64±6 s, *n*=19) and *klp6*Δ (control, 76±6 s, *n*=18; TBZ, 69±5 s, *n*=14) cells in the presence or absence of 10 µg/ml of TBZ. (C) Amplitude of kinetochore oscillations obtained with the Fourier transform method during metaphase in wild-type (control, 0.23±0.02 μm, *n*=24; TBZ, 0.14±0.015 μm, *n*=18) and *klp6*Δ (control, 0.35±0.04 μm, *n*=19; TBZ, 0.13±0.02 μm, *n*=14) cells in the presence or absence of 10 µg/ml of TBZ. (D) Global distribution of the normalized distances (nd) between Cen2 to the spindle center for each high-frame-rate time-point during metaphase in wild-type (control, 0.09±0.0002, *n*=73, 460; TBZ, 0.06±0.0002, *n*=63, 390) and *klp6*Δ (control, 0.2±0.0004, *n*=89, 101; TBZ, 0.14±0.0004, *n*=59, 997) cells. Each distance between sister kinetochores to the spindle center is normalized according to spindle size so that sister kinetochore position varies between 0 (spindle center) to 0.5 (spindle poles). The plots in B–D are violin plots, which combine a standard box plot with a density trace. Each circle represents a value in the dataset, the horizontal bar represents the mean of the distribution and the vertical bar represents s.e.m. **P<0.01, ****P<0.001 (Student's *t*-test).

To accurately quantify the variations in kinetochore movements, we determined the amplitudes and half-periods of oscillations in both cell types using Fourier analysis (Fig. 4A; Fig. S3; see Materials and Methods). The analysis revealed that the half-period (*T*_1/2_) of these oscillations was not significantly different in the absence of kinesin-8 (Fig. 4B), whereas its corresponding amplitude was increased by a factor of ∼1.5 (Fig. 4C). These results were confirmed using a second independent method to characterize the half-periods and amplitudes of chromosome oscillations (see Materials and Methods; Fig. S3). Both methods gave qualitatively similar results (Fig. S3D,E). Thus, kinesin-8 in fission yeast dampens chromosome motions by reducing their speed as observed in human HeLa cells (Jaqaman et al., 2010; Stumpff et al., 2008). Importantly, amplitudes and periods of oscillations were not significantly affected by increasing spindle size (Fig. S3F,G).

To address whether the chromosome congression defects observed in kinesin-8-deleted cells result from an increase in the amplitude of oscillations, we analyzed chromosome movements in wild-type and kinesin-8-deleted cells in the presence of low doses of TBZ. The amplitude of chromosome movements was significantly reduced in both cell types as compared to control conditions (Fig. 4C), whereas the period of oscillations was unchanged (Fig. 4B). Thus, fission yeast kinetochore oscillations are largely powered by MT depolymerization. Interestingly, chromosome alignment in the presence of TBZ was slightly improved in both wild-type and mutant cells. However, abrogating oscillations was not sufficient to fully rescue the kinetochore alignment defects observed in kinesin-8-deleted cells (Fig. 4D).

Taken together, these observations demonstrate that an increase in MT-driven chromosome oscillation is not sufficient to explain chromosome congression defects of cells deleted for kinesin-8. Thus, an alternative mechanism controls chromosome congression in fission yeast independently of MT depolymerization and chromosome oscillation.

### The presence of unattached kinetochores is not sufficient to explain the centering defects of kinesin-8 mutants

**Fig. 5.**
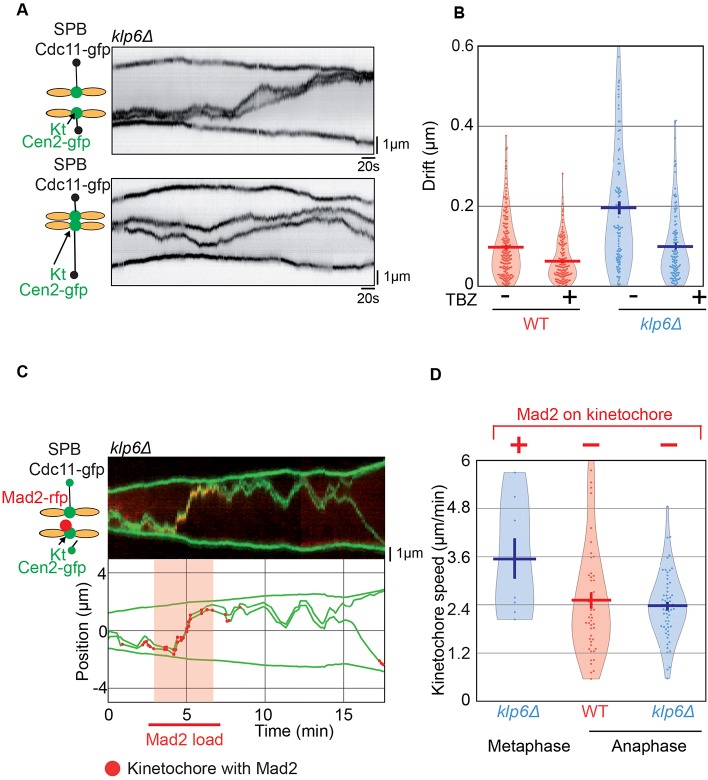
**Kinesin-8 mutants display imbalanced traction forces at kinetochores leading to chromosome detachment and instability in kinetochore positioning.** (A) Typical kymographs obtained at a high-frame rate (frames were taken every 0.1 s) of *klp6*Δ cells expressing Cen2–GFP (to mark sister centromeres) and Cdc11–GFP (to mark SPBs) during metaphase. Note the instability of kinetochore positioning in metaphase (also called drift; Fig. 4A). (B) Determination of drift amplitudes between each oscillation event during metaphase in wild-type (WT) (control, 0.1±0.006 μm, *n*=171; TBZ, 0.06±0.004 μm, *n*=141) and *klp6*Δ (control, 0.2±0.02 μm, *n*=104; TBZ, 0.1±0.008, *n*=114) cells in the presence or absence of 10 µg/ml TBZ. (C) Typical kymograph obtained at high-frame rate (frames were taken every 0.1 s) and corresponding trajectories of *klp6*Δ cells expressing Cen2–GFP (to mark sister centromeres, green), Cdc11–GFP (to mark SPBs, green) and Mad2–mCherry (to mark spindle checkpoint protein, red) during metaphase. The red circles on kinetochore trajectory and the red area illustrate the detection of Mad2–mCherry during the burst. (D) Determination of the maximum kinetochore speed at anaphase A in wild-type and *klp6*Δ cells as compared to the maximum kinetochore speed during a detachment event (as judged by the Mad2 burst) in *klp6*Δ cells in metaphase. The plots in B and D are violin plots, which combine a standard box plot with a density trace. Each circle represents a value in the dataset, the horizontal bar represents the mean of the distribution and the vertical bar represents s.e.m.

We hypothesized that imbalanced traction forces at kinetochores could lead to chromosome alignment defects by causing the appearance of drifts in kinetochore trajectories (Fig. 4A). Indeed, the analysis revealed the presence of large drifts in cells deleted for kinesin-8 as shown in Fig. 5A, and measured in Fig. 5B. Given that previous studies have revealed that kinesin-8 is required for correct MT attachment (Garcia et al., 2002a), we reasoned that these large drifts in trajectories could be due to attachment defects. To test this hypothesis, we simultaneously analyzed kinetochore dynamics and the localization of the checkpoint protein Mad2 in a *cen2–GFP mad2–mCherry* strain deleted for Klp6. As previously described, in wild-type cells Mad2 localizes diffusely around the nuclear envelope during G2. As cells enter mitosis, Mad2 relocates to a region underlying the unseparated spindle poles that colocalizes with unattached kinetochores, remaining in this area as the spindle forms (Ikui et al., 2002). Once the spindle reaches a length of between 1 and 2 μm, Mad2 no longer colocalizes with the bi-oriented kinetochores (Courtheoux et al., 2007). In the Klp6 mutant, as opposed to wild-type, we observed bursts of Mad2 appearing on the kinetochore pair just prior to these large drifts, suggesting, in this case, that there was a total loss of chromosome attachment (an example of this phenomenon is shown in Fig. 5C). In agreement with this hypothesis, kinetochore speed during such crossing was similar to kinetochore speed at anaphase indicating that chromosomes must be detaching from one of the poles (Fig. 5D). Soon after the drifts, chromosomes rapidly reattached as judged by the disappearance of Mad2 and the reappearance of kinetochore oscillations (Fig. 5C).

Thus, kinetochore detachment might explain the increased amplitude of oscillations observed in kinesin-8-deleted cells. However, these drifts (as well as Mad2 bursts) were absent in the presence of low doses of TBZ (Fig. 5B). Given that the centering defects were still observed in the presence of TBZ in kinesin-8-deleted cells, our results suggest that the role of kinesin-8 in kinetochore centering is independent of stable kinetochore attachment.

### Kinesin-8 accumulates at the plus-end of intra-nuclear spindle MTs in a length-dependent manner

**Fig. 6.**
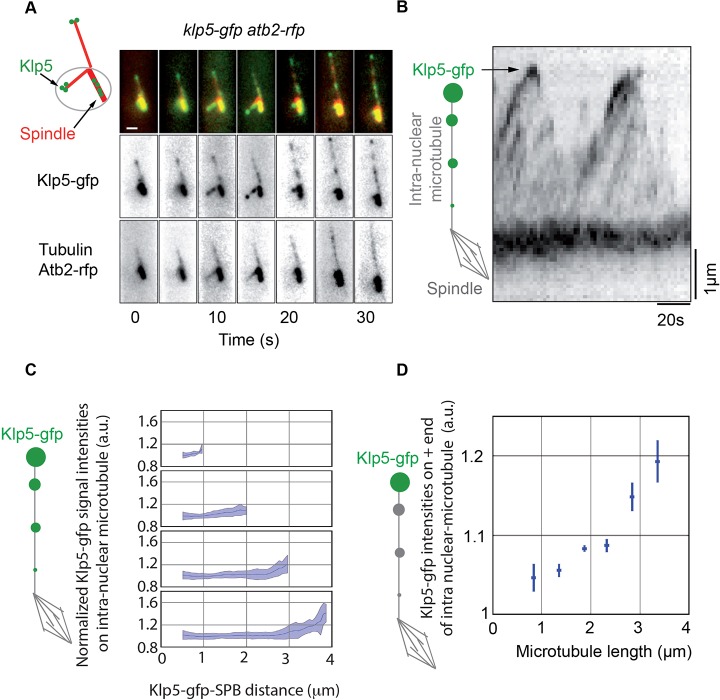
**Kinesin-8 accumulates at the tip of mitotic MTs in a length-dependent manner.** (A) Typical time-lapse fluorescence images of wild-type cells expressing Atb2–GFP (tubulin) and Klp5–GFP (frames were taken every 5 s). Scale bar: 1 μm. (B) Kymograph representation of Klp5–GFP localization on intra-nuclear MTs (note the accumulation at the plus end). Cells were blocked in G2 at the restrictive temperature, released into mitosis at 25°C and the Klp5–GFP signal was captured at a high frame rate (frames were taken every 2 s). (C) Klp5–GFP signal intensity is shown according to its position along mitotic MTs for several sizes of MT. Multiple frames were used for each respective plot and originate from 37 individual cells (from top panel to bottom panel; 0.5 to 1 µm, *n*=29; 1 to 2 µm, *n*=240; 2 to 3 µm, *n*=154; 3 to 4 µm, *n*=81). Blue lines indicate mean values and cyan shading represents standard deviations. (D) Normalized Klp5–GFP intensities at the plus end of MTs as a function of intra-nuclear MT length (0.5 to 1.0 µm, *n*=29; 1.0 to 1.5 µm, *n*=94; 1.5 to 2.0 µm, *n*=146; 2.0 to 2.5 µm, *n*= 90; 2.5 to 3.0 µm, *n*=64; 3.0 to 3.5 µm, *n*=59). The blue bars indicate s.e.m.

In order to clarify the kinesin-8-dependent mechanism at the origin of kinetochore centering, we analyzed Klp5 movements on nuclear MTs in mitosis. Although it is currently known that Klp5 translocates to the nucleus during mitosis (Unsworth et al., 2008), where it can be found at the kinetochores (Garcia et al., 2002b), the precise coordination between Klp5 movements and nuclear MT dynamics in mitosis has never been investigated. As it was not possible to visualize Klp5–GFP on individual MTs within the spindle, we instead recorded the progression of Klp5 along intra-nuclear MTs emanating from the spindle pole bodies in mitosis (Gachet et al., 2008) using a *klp5–GFP atb2–RFP* strain. During MT growth, Klp5–GFP patches rapidly moved on MTs to accumulate at the plus-end tips of intra-nuclear MTs. Then, the disappearance of Klp5–GFP patches correlated with the rapid depolymerization of MTs (Fig. 6A; Movie 3).

To quantify the accumulation of Klp5–GFP at the plus end of these MTs we performed high-frame rate acquisitions (Fig. 6B). We reported the intensity profile of Klp5–GFP along MTs for different MT length (see Materials and Methods for the normalization process). An accumulation of Klp5 was detectable at the plus-end tips of MTs in all cases, on either short (Fig. 6C, top panel) or long MTs (Fig. 6C, bottom panel). However, the maximum intensity of fluorescence at the plus end of intra-nuclear MTs increased with MT length (Fig. 6D), suggesting that kinesin-8 accumulates strongly on long MTs but less strongly on short ones during metaphase. Such behavior suggests that kinesin-8 could control traction forces at the kinetochore in a length-dependent manner, meaning that the longer is the MT, the higher its probability of catastrophe or its depolymerization rate. In agreement with this hypothesis, the length of intra-nuclear MTs was increased in cells deleted for kinesin-8 as opposed to wild type (data not shown).

Taken together, these observations suggest that kinesin-8 controls the size of MTs, and thus forces, through MT depolymerization, in a length-dependent manner.

### A length-dependent control of pulling force is sufficient for kinetochore centering and prevents the appearance of lagging chromosomes

**Fig. 7.**
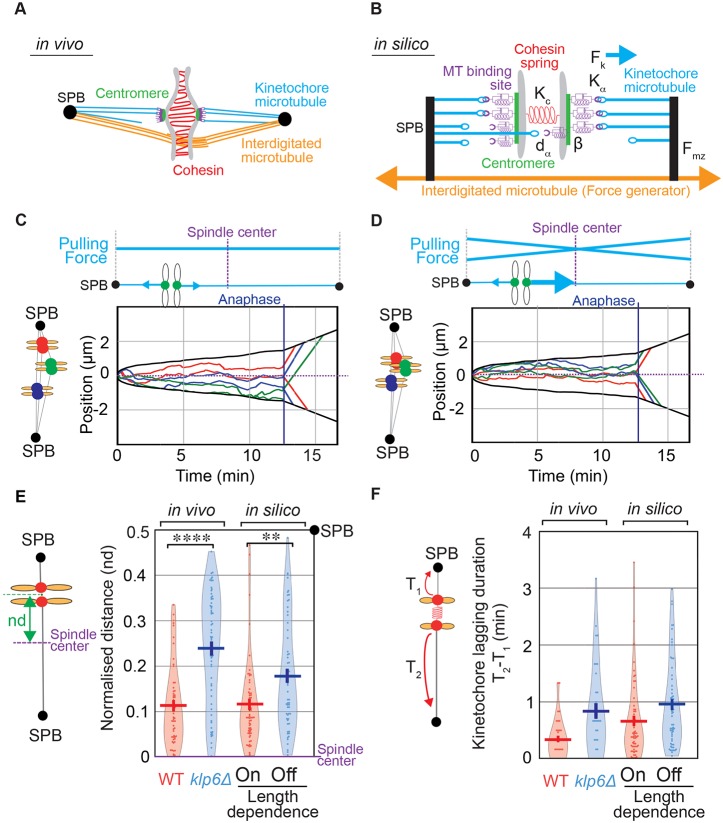
**A length-dependent pulling force centers kinetochores and prevents lagging chromosomes in a force-balance model.** (A) Schematic representation of the metaphase spindle. The two SPBs (black) are linked by overlapping interdigitated MTs (orange). The chromosome (gray) is linked to the SPBs by its centromere regions (green). The three MT-binding sites located on each kinetochore (purple) are connected to the SPBs by ktMTs (blue lines). The two sister chromatids are held together by the cohesin complex (red). (B) Biophysical representation of the metaphase spindle. The SPBs are linked by the interdigitated MT force generator (F_mz_, orange). Each MT attachment site on the kinetochore (purple) is linked to the SPB through a kinetochore MT (blue). The three MT-binding sites (purple) are associated with the chromosomes by the centromere (green) and represented by a spring and damper (purple). Cohesin between the sister chromatids (red) is modeled as a single spring linking both centromeres (K_c_). A simple stochastic process of MT attachment and detachment reproduces the directional instability. At any time, MT attachment sites (purple) attach with the frequency *k*_a_ (force *F*_k_ is ON) or detach with the frequency *k*_d_ (force *F*_k_ is OFF). This attachment and detachment process leads to an imbalance of the forces applied on the chromosome and to chromosome dynamics within the spindle. The parameter *d*_α_ (Aurora-B-like activity) modulates the probability of MT detachment as a function of the distance between the MT attachment site and the center of the kinetochore pair. Thus, the parameter *d*_α_ is defined as the spatial range of Aurora B activity. The kinetochore orientation effect parameter (β) controls the probability for a new kinetochore-MT attachment to be correct or incorrect depending on the previous attachment state of the kinetochore to the poles. When β equals 1 and the kinetochore is attached to a single spindle pole, the next attachment cannot be erroneous. When β equals 0, correct or erroneous attachments are equiprobable (the model parameters are detailed in Gay et al., 2012). (C,D) Top panels, diagrams depicting the presence (D) or absence (C) in the force-balance model of a length-dependent pulling force (see Movie 4 for animated trajectories). Lower panel, typical *in silico* trajectories of the three pairs of kinetochores (blue, red and green) and the two SPBs (black) obtained in the presence (D) or absence (C) of a length-dependence pulling force. (E) Distance (nd) between kinetochores to spindle center at anaphase onset normalized according to spindle length *in vivo* [wild type (WT), *n*=52 and *klp6*Δ, *n*=63] or *in silico* (in the presence, *n*=600 or absence of length dependence, *n*=600). (F) Kinetochore lagging time from anaphase A onset *in vivo* (wild-type, *n*=37 and *klp6*Δ cells, *n*=34) or *in silico* (in the presence, *n*=503 or absence of length dependence, *n*=451). The plots in E and F are violin plots, which combine a standard box plot with a density trace. Each circle represents a value in the dataset, the horizontal bar represents the mean of the distribution and the vertical bar represents s.e.m.

**Fig. 8.**
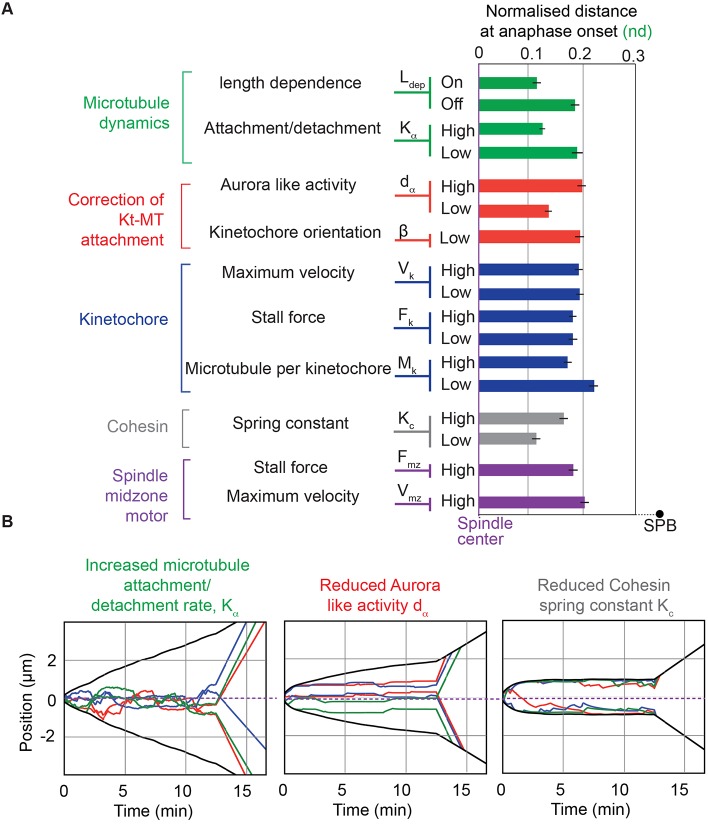
**Influence of several model parameters on kinetochore alignment.** (A) Normalized distance between sister kinetochore position to the spindle center at anaphase onset. Several model parameters were either increased (High) or decreased (Low) as compared to the default value (Table S1) to study their influence on kinetochore alignment. Simulations were obtained with the length-dependent pulling force turned off. These parameters correspond to different processes within the spindle (i.e. MT dynamics, correction of kinetochore–MT attachment, regulation of kinetochore-dependent processes, sister chromatid cohesion and regulation of spindle midzone motors). The figure shows the mean values±s.e.m. for an average number of simulations of 500. (B) Simulations showing the impact of parameters found to increase kinetochore alignment in the absence of a length-dependent mechanism. Left panel, increasing the rate of attachment and/or detachment, *K*_α_, had a dramatic effect on mitotic progression, such as failure to maintain spindle size in metaphase or presence of unattached kinetochores. Middle panel, the parameter *d*_α_, which represents the typical range of action of Aurora B (red) favors kinetochore alignment when it is decreased. However, in this condition, kinetochore attachments are hyperstabilized, kinetochores exhibit almost no dynamicity and merotelic kinetochore attachment is frequent (see Gay et al., 2012). Right panel, diminishing the parameter *k*_c_ (the cohesin spring constant) also seems to favor kinetochore alignment. However, this phenotype is due to the unrealistic inter-kinetochore distance reaching the entire spindle length (about 2 µm) while the *in vivo* value only equals 0.5 µm. In this condition, each chromatid is pulled towards a pole and the position of the sister kinetochores corresponds to the spindle center.

Our work suggests that kinesin-8 motors could act as regulators of force balance exerted on the bi-oriented chromosomes according to their position within the spindle. To test this hypothesis, we explored *in silico* the mechanisms of sister kinetochore centering during mitosis. We originally designed a force-balance model of mitosis that describes global spindle dynamics and predicts chromosome segregation defects (Gay et al., 2012). In this model, summarized in Fig. 7A,B, kinetochore pair breathing and oscillatory movements were modeled by a series of stochastic events of attachment and detachment at each MT attachment site, the pulling force being ‘on’ when a MT is attached and ‘off’ when it detaches. The overall pulling forces exerted on the outer side of the kinetochores are then balanced at their inner side by the cohesin retraction force. At the poles, an additional pushing force exerted on the interdigitated MTs tends to elongate the spindle and prevents its collapse (Courtheoux et al., 2009; Gay et al., 2012). In order to mimic the effect of Klp5 on the dynamics of kinetochores, we compared kinetochore centering in the absence (Fig. 7C) or presence (Fig. 7D) of a length-dependent pulling force by assuming the following hypothesis. If the catastrophe frequency or the depolymerization rate is higher for long kinetochore MTs than for short ones, then the force exerted on kinetochores attached to long MTs is on average higher than on short ones. Therefore, the modeled pulling forces on each attachment site were modulated by a spatial factor proportional to the distance between the kinetochore position and the spindle pole (see Materials and Methods and Table S1 for a description of model parameters). The value of this factor was set to reflect the distribution of kinetochore positions at anaphase onset. Typical examples of simulated trajectories with the length-dependence pulling force turned on and off are shown in Fig. 7B,D. We observed that the length-dependent pulling force faithfully reproduced the positioning of kinetochores throughout metaphase, as observed *in vivo* (Fig. 7D). Quantitative analysis confirmed that the presence of this pulling force was sufficient to reproduce kinetochore alignment in metaphase and prior to anaphase,whereas its removal mimicked the effect of kinesin-8-deletion (Fig. 7D,E). Previous work has shown that the number of lagging chromosomes at anaphase increases in *klp5Δ* cells, whereas the number of mis-segregations remained low (Sanchez-Perez et al., 2005). We used our model to evaluate the role of the centering mechanism in preventing chromosome segregation defects. As expected, *in vivo*, we found that the delay between the arrivals of the two sister chromatids at their respective pole was on average increased in kinesin-8 mutant compared to WT (Fig. 7F). The values obtained were comparable to *in silico* data when the length dependence was turned on or off (Fig. 7F). However, our model predicts that the rate of chromosome mis-segregation (two Cen2 signals at the same pole) is very low in both conditions, which is consistent with what has been reported *in vivo* (Sanchez-Perez et al., 2005; data not shown). Importantly, we modified other model parameters (including kinetochore attachment and detachment parameters, and force and velocity characteristics of pulling motors or cohesin stiffness) to evaluate their influence on chromosome alignment (Fig. 8A). Interestingly, only the addition of the length-dependent process substantially increased kinetochore centering during metaphase (Fig. 8A) without introducing aberrant mitotic phenotypes in simulations (Fig. 8B).

Taken together, our results suggest that a length-dependent pulling force is necessary and sufficient to align chromosomes and to prevent the appearance of lagging kinetochores. Our observations also imply that lagging chromosomes in kinesin-8 mutants are not caused by defective kinetochore attachment but rather due to kinetochore mis-alignment at anaphase onset.

## DISCUSSION

Our study reveals the basic mechanisms required to align chromosomes in fission yeast. Chromosome alignment before anaphase onset relies on two discrete steps. First, during phase 1 (prophase), kinetochores congress between the two spindle poles through an active mechanism. Second, during phase 2 (pro-metaphase and metaphase), kinetochores maintain this alignment at the spindle midzone while oscillating. Kinetochore oscillatory movements have been previously characterized in mammalian cells (Gardner et al., 2005; Jaqaman et al., 2010; Stumpff et al., 2008; Vladimirou et al., 2013) and in budding yeast (Pearson et al., 2001), but their role in chromosome alignment is unclear. Our work reveals that chromosome oscillations (i.e. triggered by MT depolymerization) is dispensable for chromosome centering because the suppression of oscillations with low doses of TBZ had no impact on kinetochore alignment throughout metaphase. In fission yeast, it is likely that kinetochore oscillations are mainly triggered by MT depolymerization. In agreement with this finding, the speed of kinetochores moving poleward (MT depolymerizing) is largely reduced in the presence of low doses of TBZ whereas the speed of kinetochores moving in an anti-poleward manner (polymerizing MTs) is barely unchanged (data not shown).

Kinesin-8 is emerging as one of the most important motor proteins that participate in the correct distribution of forces within the spindle. In fission yeast, kinesin-8 is required not only in phase 1 for chromosome congression but also in phase 2 to maintain stable kinetochore positioning. We favor a model where kinesin-8 controls the establishment of kinetochore centering (and thus controls the force balance at the kinetochore) to prevent kinetochore detachment at the spindle poles. In agreement with this hypothesis, we observed that kinetochore detachment generally occurs when chromosomes are located near the spindle poles (data not shown). The accumulation of kinesin-8 at the plus-ends of MTs and its function in controlling the size of intra-nuclear MTs might be reminiscent of what has been reported *in vivo* in interphase fission yeast cells (Tischer et al., 2009) and *in vitro* for the budding yeast kinesin-8 homolog, Kip3p or the human homolog Kif18A (Mayr et al., 2007). The role of length-dependent forces in kinetochore centering might be analogous to length-dependent pulling forces required for spindle centering in vertebrates (Mitchison et al., 2012; Wühr et al., 2009), except that kinesin-8 would play a role as a centering agent able to measure MT length in order to place the kinetochore at the spindle center.

Our study also reveals that an increase in MT-driven chromosome oscillation is not sufficient to explain the centering defects of kinesin-8 mutants given that abolishing chromosome oscillation does not restore alignment. It is thus tempting to speculate that the early stage of congression in fission yeast is reminiscent of the role of kinesin-7 (CENP-E) in the sliding of chromosomes along spindle MTs (Cai et al., 2009; Kapoor et al., 2006). Accordingly, *in vitro* studies suggest that *S. pombe* kinesin-8 might have plus-end-directed motor activities and share some properties with the kinesin-7 family (Grissom et al., 2009).

The role of kinesin-8 in kinetochore positioning can be mathematically reproduced by adapting the pulling force applied at kinetochores according to its position within the spindle. Indeed, a non-uniform pulling force is sufficient to align and maintain kinetochore alignment throughout metaphase. Similarly, modeling a non-uniform distribution of MT plus ends across the spindle is also sufficient to align chromosomes (data not shown), but further work would be necessary to discriminate between these two hypotheses. By contrast, increasing or decreasing other model parameters such as the kinetochore attachment or detachment rate (i.e. frequency of kinetochore movements) is not sufficient for chromosome centering. Our model makes no hypothesis on the nature of the molecular motor involved in this centering mechanism or the origin of this length-dependence mechanism. However, *in vivo* accumulation of kinesin-8 during mitosis according to MT length is consistent with a modulation of the frequency rate of catastrophe (Gardner et al., 2008; Tischer et al., 2009; Varga et al., 2009) and as observed by electron microscopy, the size of MTs in the spindle is not uniform (Ding et al., 1993). Our model is not considering that chromosome alignment might also be influenced by correlated movements of non-sister kinetochores as recently described in human cells (Vladimirou et al., 2013). However, the spatial organization of chromosomes might be important for chromosome congression (Kitajima et al., 2011; Magidson et al., 2011), especially considering that physical links exist between telomeres of chromosomes in mitosis (Reyes et al., 2015).

Either way, our work demonstrates that a gradient of force is sufficient to align chromosomes, to maintain this alignment and to prevent the appearance of lagging chromosomes at anaphase onset. Given that the biological function for chromosome movements in mitosis remains elusive, our study provides the basis to understand this important question.

## MATERIALS AND METHODS

### Cell culture

Media, growth, maintenance of strains and genetic methods were as previously reported (Moreno et al., 1991). Cells were grown at 25°C in yeast extract before mounting on an imaging chamber. The strains used in this study are listed in Table S2.

### Live-cell imaging

Live-cell microscopy was performed on an imaging chamber (CoverWell PCI-2.5; Grace Bio-Labs, Inc.) filled with 1 ml of 2% agarose in minimal medium and sealed with a 22×22 mm glass cover-slip. The temperature was maintained at 25°C during acquisitions. Images were acquired from an inverted wide-field microscope (Nikon Eclipse TI) equipped with a Neo sCMOS camera (Andor Technology Ltd), a LED light source (Lumencor Spectra) and a 100× objective (1.45 NA). Images were recorded using the free open-source Micro-Manager software (Edelstein et al., 2010).

For quantitative analysis of kinetochore alignment (using Cen2–GFP or Ndc80–GFP), images were acquired every 10 s with 10 Z-section of 300 nm at each time step. It has been previously reported that the distance between Cen2–GFP and Ndc80–GFP spots is ∼120 nm, less than the typical size of these spots (Gay et al., 2012). Thus, in this study the term kinetochores of chromosome 2 has often been used instead of pericentromeric region of chromosome 2.

For high-frame rate acquisitions (analysis of chromosome oscillations), images were acquired with a single *Z*-section and a time step of 100 ms. The *Z* position was manually modified during acquisitions to maintain the focus on the spindle. In thiabendazole (TBZ) assays, cells were imaged in minimal medium supplemented with 10 µg ml^−1^ TBZ (from a stock solution of 10 mg ml^−1^ in DMSO). Cells were incubated for 30 min at 25°C before image acquisitions.

For *cen2–GFP*, *cdc11–GFP* and *mad2–RFP* acquisitions, images were acquired every 7 s with 3 Z-sections of 600 nm. To image Klp5–GFP on intra-nuclear MTs, images were acquired every 2 s with 3 Z-sections of 300 nm; Cdc11–CFP signal was acquired every 30 s. To record the position of kinetochores at the very beginning of mitosis, cells with Ndc80–GFP and Cdc11–CFP markers were used (Tournier et al., 2004). Acquisitions were made every 5 s with 5 Z-sections of 400 nm each.

### Image analysis

Image analysis was performed using Fiji software (Schindelin et al., 2012), Python scripts in the scientific python ecosystem [SciPy library (Oliphant, 2007)] and custom software developed in the lab. All source code used in this paper is open source and freely available at https://github.com/hadim/spindle_tracker.

#### Peak detection and tracking

Cdc11–GFP (SPB) and Cen2–GFP (centromeres of chromosome 2) spots were first detected after a maximum *Z*-projection of images using LoG detector from TrackMate (Fiji plugin). Then, tracking was performed with custom software developed in Python. To link these spots with time, we assumed that the most distant spots were the SPBs, and that the two remaining were the centromeres. Each trajectory was then projected on the spindle axis defined by the two SPBs.

#### Quantification of fluorescence signal

Intra-nuclear MTs (iMt) were manually detected. For each iMt, a mean profile of Klp5–GFP intensity of 4-pixel width was computed. For normalization, intensities were divided by the median intensity of the profile. Then, for each range of length, the intensities of all iMts were averaged.

#### Quantification of MT dynamics

MT dynamics quantification was performed with a custom ImageJ macro working as follows. The image stack was smoothed using the ‘Gaussian Blur’ filter before applying a maximum *Z*-projection. The projected images were then filtered in the Fourier space to remove wavelengths larger than 12 pixels (0.8 µm) and smaller than 3 pixels (0.2 µm). Regions of interest (ROIs) were defined around individual interphasic MTs and the ‘Triangle’ auto-thresholding algorithm was applied in these regions. Binary images were then skeletonized to produce unidimensional shapes whose length *L* was extracted at each time-step. The curve *L*(*t*) is manually divided into growing and shrinking periods, fitted with a linear function to extract growing and shrinking rates.

#### Kymographs

Kymograph representations were performed with a custom Fiji macro available upon request (https://github.com/hadim/fiji_tools/blob/master/macros/AutoInstall/custom-macros.ijm#L45).

### Characterization of amplitudes and periods of oscillations

#### Fast Fourier transform analysis

To characterize kinetochore oscillations, we performed Fast Fourier transform (FFT) analysis on each tracked trajectory of chromosome 2 (middle of the two Cen2–GFP spots; blue line in Fig. S3A). To convert the power spectrum into an amplitude of movement (in µm), we normalized it by the length of the signal and multiplied by two (half of the spectrum is removed so energy must be preserved). Peaks were detected as local maxima of the FFT curve. Peaks with frequency lower than 5×10^−3^ Hz were excluded (high-pass filter). The peak of highest amplitude was used for the characterization of the oscillatory movement.

#### Detection of local maxima

To characterize kinetochore oscillations, we first smoothed kinetochore trajectories by fitting a spline function (red line in Fig. S3C). The middle time-point between the two peaks of two consecutive local extrema defines the start of a semi-period and the following middle time-point indicates the end of the semi-period (Fig. S3C).

### Statistical analysis

Errors mentioned in text and figures indicate the s.e.m. except when specified. The violin plots used in this paper combines a standard box plot with a density trace. Each circle represents a value in the dataset and the horizontal bar represents the mean of the distribution. Statistical tests were performed using a Student's *t*-test (Python library SciPy, scipy.stats.ttest_ind). Statistical significance is defined as follows: NS, *P*>0.05, *P<0.05, **P<0.01, ***P<0.001 and ****P<0.001.

### Modeling

The source code of the mitotic model (Gay et al., 2012) is available under an open source license at https://github.com/bnoi/kt_simul.

#### General assumption

As previously described, Klp5 and Klp6 re-localize at the spindle midzone during anaphase (West et al., 2001). Thus, in the simulations the length-dependent mechanism described below was turned off during anaphase.

#### Length-dependent pulling force

In the initial model (Gay et al., 2012), the pulling force (*F*) applied on single attachment sites follows a linear force–velocity relationship:
(1)
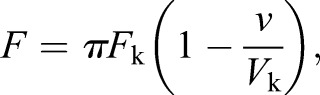
where *F*_k_ and *V*_k_ are the stall force and the motor maximum velocity, *v* is the speed of the attachment site and π is the attachment state (equal to 1 when the site is attached to the correct pole, −1 when it is attached to the opposite pole and 0 when it is detached).

The length-dependent mechanism was implemented by adding a prefactor *L*_dep_ to this pulling force:
(2)
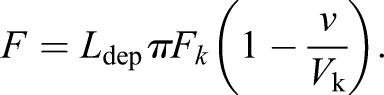
*L*_dep_ is calculated according to the actual distance between the attachment site and its corresponding pole (*d*_site-pole_) following a linear relationship:

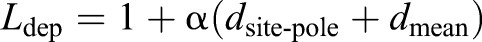
Where α is a free parameter governing the strength of the relation between the distance *d*_site-pole_ and force magnitude. *d*_mean_ is the average *in vivo* distance measured between the kinetochore attachment site and the pole during metaphase. Finally, *d*_site-pole_ is the actual distance between the kinetochore attachment site and the pole. In this study we used *d*_mean_=1 µm and α=0.2. The value of α is optimized to reproduce kinetochore centering as observed *in vivo*.
